# Cardiac Surveillance for Early Detection of Late Subclinical Cardiac Dysfunction in Childhood Cancer Survivors After Anthracycline Therapy

**DOI:** 10.3389/fonc.2021.624057

**Published:** 2021-05-14

**Authors:** Rosaria Sofia, Veronica Melita, Antonio De Vita, Antonio Ruggiero, Alberto Romano, Giorgio Attinà, Lisa Birritella, Priscilla Lamendola, Antonella Lombardo, Gaetano Antonio Lanza, Angelica Bibiana Delogu

**Affiliations:** ^1^ Unit of Pediatrics, Pediatric Cardiology, Department of Woman and Child Health and Public Health, Fondazione Policlinico Universitario A. Gemelli IRCCS, Rome, Italy; ^2^ Catholic University of The Sacred Heart, Rome, Italy; ^3^ Department of Cardiovascular and Thoracic Sciences, Fondazione Policlinico Universitario A. Gemelli IRCCS, Rome, Italy; ^4^ Pediatric Oncology Unit, Department of Woman and Child Health and Public Health, Fondazione Policlinico Universitario A. Gemelli IRCCS, Rome, Italy

**Keywords:** childhood cancer, anthracycline, late-onset cardiotoxicity, two-dimensional echocardiography, tissue Doppler imaging, speckle tracking echocardiography, three-dimensional echocardiography

## Abstract

**Background:**

In childhood cancer survivors (CCSs) anthracycline-related cardiotoxicity is an important cause of morbidity and late mortality, but the optimal modality of cardiac surveillance still remains to be defined. The aim of this study was to assess whether non-invasive echocardiography-based functional cardiac measures can detect early subclinical myocardial changes in long-term pediatric cancer survivors who received anthracycline therapy.

**Methods:**

Twenty anthracycline-treated long-term CCSs and 20 age, sex, and body surface area matched healthy controls were enrolled in this study. Among cancer survivors, mean age at diagnosis was 6.5 ± 4.4 years, and the mean cumulative anthracycline dose was 234.5 ± 87.4 mg/m^2^. All subjects underwent a comprehensive functional echocardiographic protocol study including two-dimensional echocardiography (2D Echo), tissue Doppler imaging (TDI), speckle tracking (STE) and three-dimensional echocardiography (3D Echo). Patients were studied at a mean follow-up time of 6.5 ± 2.8 years from the end of therapy.

**Results:**

No significant differences in two-dimensional left ventricle ejection fraction (LVEF), diastolic parameters and speckle tracking (STE)-derived myocardial strain were observed between patients treated with anthracyclines and controls. Myocardial performance index was significantly prolonged (p = 0.005) and three-dimensional LVEF was significantly reduced (p = 0.002) in CCSs compared to controls, even though most values were within the normal range. There were no significant correlations between 2D, STE, and 3D echocardiographic parameters and age at diagnosis or duration of follow-up. No significant differences in echocardiographic parameters were found when stratifying cancer patients according to established risk factors for anthracycline cardiomyopathy.

**Conclusions:**

This study found significantly reduced three-dimensional LVEF in CCSs compared with controls, despite no significant differences in two-dimensional LVEF and longitudinal strain values. These findings suggest that long-term CCSs who had received anthracycline therapy may be found to have subclinical features of myocardial dysfunction. However, further studies are needed to demonstrate the validity of new imaging techniques, including STE and 3D Echo, to identify patients at risk for cardiomyopathy in the long-term follow-up of CCSs.

## Introduction

Advances in treatment strategies for childhood cancer have resulted in a significant improvement in survival (1, 2), but there is growing concern about the long-term side effects of therapy (3, 4). Among the most used chemotherapy drugs, anthracycline is a cornerstone in the treatment of several neoplastic diseases, including acute lymphoblastic leukemia, rhabdomyosarcomas, neuroblastoma, acute myeloid leukemia, Hodgkin lymphoma, osteosarcoma, and Ewing’s sarcoma. However, the use of these drugs is strongly conditioned by their short and long term cardiac toxicity (4–6)

Left ventricle (LV) dysfunction and heart failure (HF) are the most concerning cardiovascular complications of cancer therapies and could increasingly become a main cause of morbidity and death of childhood cancer survivors s (CCSs) (7, 8).

The early detection of cardiotoxicity is often challenging, due to a long subclinical phase of LV dysfunction; however, an early diagnosis of cardiovascular disease in these patients would be crucial as it could allow a timely initiation of cardioprotective therapies that might delay myocardial remodeling and prevent LV cardiomyopathy.

Because of its widespread availability and safety, two-dimensional echocardiography (2D Echo) is standardly utilized in monitoring cancer patients, although it is not always adequate to detect an early, clinically asymptomatic stage of disease (9). Recently, new imaging approaches, including tissue Doppler imaging (TDI), speckle tracking echocardiography (STE), and three-dimensional echocardiography (3D Echo), have been utilized for early detection of asymptomatic LV cardiac dysfunction (10).

The aim of this study was to assess whether advanced non-invasive echocardiography-based methods can be helpful in detecting early subclinical myocardial dysfunction in childhood anthracycline-exposed cancer survivors.

## Materials and Methods

### Study Population

This is an observational case–control study investigating 20 CCSs who received anthracycline therapy compared with 20 healthy subjects matched according to age, sex, and body surface area.

CCSs patients were followed at the Pediatric Oncology Unit of the Fondazione Policlinico Universitario A. Gemelli IRCCS, Rome, Italy and were evaluated between October 2019 and February 2020. All patients received anthracycline therapy by a central line, with anthracycline infusion lasting 4 h.

Inclusion criteria for the study were: completion of anthracycline therapy for more than 3 years; cumulative anthracycline dose <360 mg/m^2^; no symptoms of HF and normal global LV systolic function [defined as a LV ejection fraction [LVEF] ≥55%, and a LV shortening fraction (LVSF) ≥28%] at the last standard echocardiogram. Exclusion criteria included a history of congenital cardiac defects or any other cardiac disease; mediastinal/chest radiotherapy; bone marrow or staminal cells transplantation; signs and/or symptoms of acute and early cardiotoxicity. Healthy subjects were children referred to our Pediatric Cardiology Unit for complaints such as chest pain or palpitation, who were found to have normal cardiac examination, electrocardiogram, and standard transthoracic Doppler echocardiography.

Prior to the participation, the patients and their parents provided signed consent forms after being informed about the aim of the project as foreseen by the Italian Law on Privacy and the Safeguarding of the Sensitive Data (D.LGS n196, 2003). The study was carried out in accordance with the Helsinki declaration of human rights. Approval by the Ethics Committee was not necessary since echocardiography is recommended as the primary cardiomyopathy surveillance modality for assessment of left ventricular systolic function in follow-up of survivors treated with anthracyclines.

### Clinical Characteristics

Demographic characteristics, blood pressure (BP), heart rate (HR), body mass index (BMI), and body surface area (BSA) were collected from all subjects. Type of malignancies, age at diagnosis, time since the last anthracycline dose, cumulative anthracycline dose, non-modifiable risk factors (female sex, age <5 years at diagnosis, concomitant administration of other chemotherapeutic agents, concomitant radiotherapy, cumulative anthracycline dose >250 mg/m^2^, renal failure), and modifiable cardiovascular risk factors (hypertension, obesity, smoking, sedentary habit) were collected from CCSs patients.

### LV Echocardiographic Study

All the subjects underwent a comprehensive functional echocardiographic protocol study including two-dimensional echocardiography (2D Echo), tissue Doppler imaging (TDI), speckle tracking (STE), and three-dimensional echocardiography (3D Echo).

All echocardiographic studies were performed by an experienced physician (a cardiologist expert in non-invasive imaging, together with a pediatric cardiologist), using a Philips EPIQ 7C^®^ ultrasound system with a Philips X5-1 probe (Philips Medical System, Andover, Massachusetts, USA). Standard imaging planes were acquired according to current guidelines and recording three beat loops saved in DICOM format and stored in a workstation. These digital loops were interpreted by the same physicians, together, and both were blind to the subject’s group.

First, we assessed LV morphology and function by M-mode and 2D echo. Standard echocardiographic parameters included LV end-diastolic (LVEDD) and end-systolic (LVESD) diameters, LV end-diastolic (LVEDV) and end-systolic (LVESV) volumes, interventricular septum (IVS) and posterior wall (PW) thickness. Measurements were indexed for BSA. As surrogates of LV systolic function, we determined the LVSF on M-mode imaging, LVEF by biplane Simpson’s calculation, the mitral annular plane systolic excursion (MAPSE) and the myocardial performance index (MPI). MPI is an index that incorporates both systolic [isovolumic contraction time (IVCT) and LV ejection time (LVET)] and diastolic [isovolumic relaxation time (IVRT)] time intervals. It is, therefore, an expression of both global systolic and diastolic LV function and is calculated with the following formula: IVCT + IVRT/LVET. An MPI ≤0.40 is considered to be normal (11). LV dimension and function assessment was performed according to the recommendations of the American Society of Echocardiography (ASE) and the European Association of Cardiovascular Imaging (EACVI) (12, 13).

Diastolic function was assessed following the recommendations of the last guidelines (14). The following parameters were obtained by pulsed wave Doppler and TDI: 1) E/A wave ratio, *i.e.*, the ratio between the peak of early diastolic Doppler mitral blood flow velocity (E wave) and the peak of late diastolic Doppler mitral blood flow velocity (A wave); 2) septal and lateral E/E′ wave ratio, *i.e.*, the ratio between the E wave peak and the peak of the early diastolic myocardial velocity (E′ wave) on tissue Doppler imaging.

Then, STE was used to quantify myocardial deformation (“strain”) as an index of myocardial function (15). Peak regional and global LV longitudinal strain was obtained from the apical two-, three- and four-chamber views. The best single cardiac cycle was selected for the analysis. After placing two mitral annular points and one apical point for each view, borders were traced semi-automatically and, if unsatisfactory, manually edited. The software automatically generated deformation curves and a “bull’s eye” diagram as a topographic intuitive representation of the values obtained for each of the 17 myocardial segments (16). According to vendor-specific indications, strain values were considered normal if > 20%.

Lastly, we evaluated three-dimensional LV volumes and LVEF. 3D Echo is a novel echocardiographic technology that is based on the acquisition of “volumes” containing heart structures (the so-called 3D datasets). 3D measurements of volumes and LVEF have been proved to be more accurate and reproducible compared to standard 2D Echo and similar to those obtained with cardiac magnetic resonance (CMR) (17, 18). For each subject, three datasets were acquired and stored. The best single-beat volumetric dataset was selected for the analysis. Once identified the end-diastolic and the end-systolic frames, four mitral annular points and one apical point were placed as markers. Then LV endocardial borders were automatically traced by the software and manually adjusted when indicated.

### Statistical Analysis

Continuous variables are reported as means and standard deviations, while categorical variables as numbers and percentages. The analysis of variance test was used to compare continuous variables, whereas nominal variables were compared by Fisher exact test. Correlation analysis was done by Pearson’s test. Statistical significance was set at a p value <0.05.

## Results

The main demographic and clinical characteristics of the two groups are summarized in **Table 1**. As expected, no differences were observed for age, sex and anthropometric data.

**Table 1:** Clinical characteristics of CCSs and healthy subjects.

Clinical characteristics	CCSs	Healthy controls	p value
**Age (years)**	13.20 ± 2.78	12.45 ± 2,91	0.410
**Male [n (%)]**	11 (55)	13 (65)	0.531
**Weight (kg)**	50.90 ± 15.16	56.10 ± 18.66	0.457
**Height (cm)**	155.05 ± 15.58	156.70 ± 18.77	0.814
**BMI**	20.69 ± 3.56	20.47 ± 4.59	0.867
**BSA (m²)**	1.44 ± 0.26	1.49 ± 0.28	0.610

Data about the neoplastic disease and treatment of CCSs patients are summarized in **Table 2**. As shown, age at cancer diagnosis was 6.50 ± 4.39 years; the time from the completion of chemotherapy was 6.50 ± 2.74 years, whereas the cumulative anthracycline dose was 234.50 ± 87.38 mg/m^2^.

**Table 2:** Specific characteristics of CCSs group.

CCS group	
**Age at diagnosis (years ± SD)**	6.50 ± 4.39
**Years since last anthracycline dose (years ± SD)**	6.50 ± 2.74
**Cumulative anthracycline dose (mg/m^2^ ± SD))**	234.50 ± 87.38
**Type of malignancies**	**N (%)**
**Acute lymphoblastic leukemia**	8 (40)
**Hodgkin lymphoma**	3 (15)
**Non-Hodgkin lymphoma**	3 (15)
**Ewing sarcoma**	5 (25)
**Neuroblastoma**	1 (5)

Acute lymphoblastic leukemia was the most common form of disease, followed by Hodgkin lymphoma, non-Hodgkin lymphoma and Ewing’s sarcoma; only one patient was affected by neuroblastoma. At the time of the present study, all CCSs were asymptomatic and did not refer any physical limitation.

## Two-Dimensional Echocardiography and Tissue Doppler Imaging

Conventional echocardiographic parameters are shown in **Table 3**.

**Table 3:** Conventional echocardiographic assessment.

Parameter (Mean ± SD)	CCS (20)	Healthy Controls (10)	p value
**FS, %**	34.20 ± 4.29	38.80 ± 7.26	0.027
**Biplane Simpson’s, EF %**	64.90 ± 3.76	65.30 ± 4.42	0.810
**EDD/BSA (mm/m^2^)**	29.55 ± 4.03	29.90 ± 6.74	0.856
**ESD/BSA (mm/m^2^)**	19.65 ± 3.39	18.40 ± 3.92	0.374
**IVSd/BSA (mm/m^2^)**	5.20 ± 1.13	5.60 ± 1.42	0.448
**PWd/BSA (mm/m^2^)**	5.00 ± 1.15	5.50 ± 1.39	0.337
**LVM/BSA (g/m^2^)**	63.70 ± 12.15	74.20 ± 9.70	0.192
**EDV (ml)**	72.60 ± 19.80	81.70 ± 28.30	0.378
**ESV (ml)**	25.80 ± 7.80	29.00 ± 11.80	0.452
**MAPSE (mm)**	16.40 ± 2.60	17.70 ± 1.49	0.157
**E (cm/s)**	98.10 ± 15.24	105.40 ± 24.27	0.328
**A (cm/s)**	63.10 ± 13.78	53.70 ± 15.21	0.103
**E/A ratio**	1.61 ± 0.42	2.05 ± 0.69	0.046*
**IVCT (ms)**	79.83 ± 16.50	67.90 ± 9.48	0.046*
**IVRT (ms)**	94.00 ± 31.94	76.10 ± 11.31	0.101
**ET (ms)**	277.38 ± 28.68	290.40 ± 19.51	0.214
**MPI**	0.62 ± 0.14	0,49 ± 0,03	0.005*
**E’ L (cm/s)**	18.47 ± 3.34	20.93 ± 4.55	0.110
**E/E’ L**	5.76 ± 1.45	4.85 ± 0.63	0.069
**E’ M (cm/s)**	12.42 ± 1.74	12.80 ± 1.60	0.573
**E/E′ M**	7.98 ± 1.43	7.00 ± 2.01	0.136

FS, fractional shortening; EF, ejection fraction; EDD, end-diastolic dimeter; ESD, end-systolic diameter; EDV, end-diastolic volume; ESV, end-systolic volume; E, early diastolic mitral inflow velocity; A, late diastolic mitral inflow velocity; IVRT, isovolumic relaxation time; IVCT, isovolumic contraction time; ET, ejection time; MPI, myocardial performance index; E’, early diastolic mitral annulus velocity; E/E’, ratio of early (E) mitral Doppler peak flow to early diastolic myocardial velocity (E′). *represent the value < 0.05 (that is the statistical significant value).

No significant differences in LV dimensions and volumes were found between the two groups. SF was slightly lower in CCS patients compared to controls, although values were still within the normal range (34.20 ± 4.29 *vs* 38.80 ± 7.26, respectively; p = 0.027). Furthermore, there was no significant difference in LVEF.

Global myocardial function evaluated through MPI resulted significantly lower in CCS patients than in controls (0.62 ± 0.14 *vs* 0.49 ± 0.03, respectively; p = 0.005). The difference was related to a significant increase in IVCT in patients (79.83 ± 16.50 *vs* 67.90 ± 9.48, respectively; p = 0.046), whereas there were no significant differences in LVET and IVRT between the two groups.

**Figure 1:**
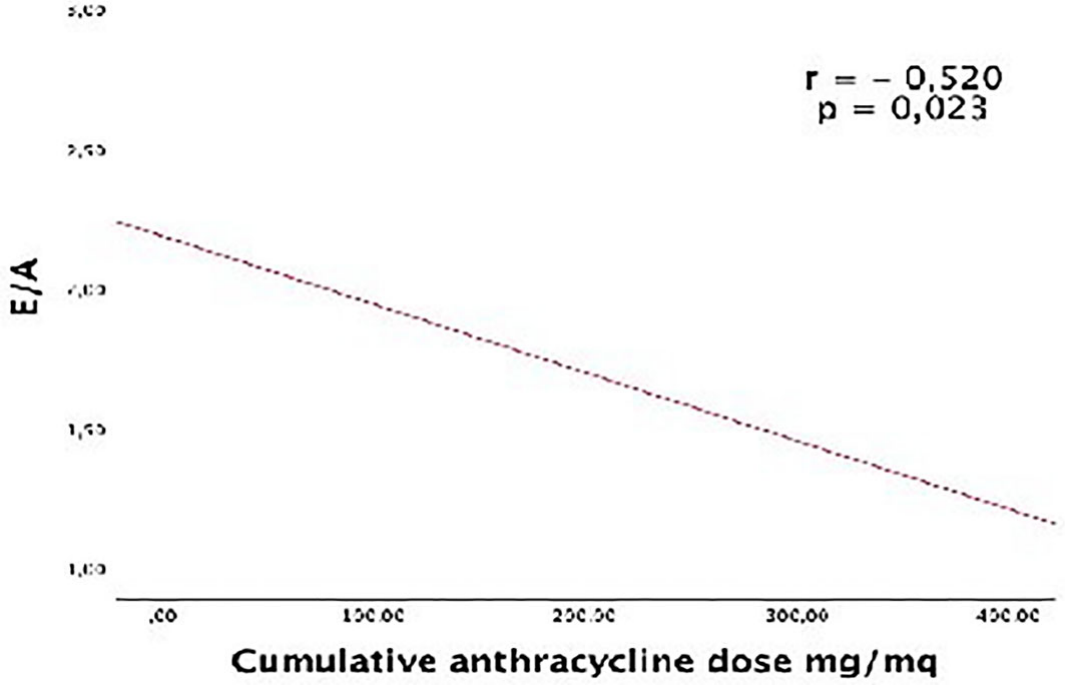
Correlation between the E/A ratio and the cumulative anthrax cycline dose.

Diastolic parameters were normal in both groups, but the E/A ratio was significantly lower in CCS patients compared to controls (1.61 ± 0.42 *vs* 2.05 ± 0.69, respectively; p = 0.046). In CC patients, an inverse correlation was found between the E/A ratio and the cumulative anthracycline dose (r = −0.520; p = 0.023; **Figure 1**).

### Speckle Tracking Imaging and 3D Echocardiography

Comparisons between CCSs and controls of STE parameters and 3D Echo parameters are shown in **Tables 4** and **5**, respectively.

**Table 4:** Left ventricular strain characteristics measured by 2D speckle-tracking echocardiography among childhood cancer survivors and controls.

STE parameters (Mean ± SD)	CCS	Healthy Controls	P value
**GLS 4CH %**	22.17 ± 2.14	21.88 ± 1.85	0.718
**GLS 3CH %**	21.54 ± 2.61	21.12 ± 2.39	0.669
**GLS 2CH %**	22,58 ± 2,90	22,52 ± 3,00	0.958
**Mean GLS**	22,12 ± 1,86	21,99 ± 1,88	0.859

GLS, global longitudinal strain; 4Ch, apical-4 chambers view; 3Ch, apical-3 chambers view; 2Ch, apical-2 chambers view.

**Table 5:** Left ventricular assessment measured by RT-3D echocardiography among childhood cancer survivors and controls.

RT-3D parameters (Mean ± SD)	CCS	Healthy Controls	P value
**EDV (ml)**	73.50 ± 25.99	84.07 ± 30.14	0.328
**ESV (ml)**	26.63 ± 9.88	25.76 ± 11.74	0.832
**EF %**	63.93 ± 3.87	69.83 ± 5.75	0.002*

EDV, end-diastolic volume; ESV, end-systolic volume; EF, ejection fraction. *represent the value < 0.05 (that is the statistical significant value).

Values of global strain on STE were normal in both groups, with no statistically significant differences. Furthermore, when stratifying for each segment, no differences were found in terms of regional longitudinal strain.

On 3D Echo imaging, LVEF was found significantly lower in CCSs compared with healthy controls (63.93 ± 3.87 *vs* 69.83 ± 5.75; p = 0.002), although values were within the normal range in all subjects. No significant differences in LV 3D volumes were found comparing patients and control group.

Finally, in CC patients there were no significant correlations between echocardiographic parameters (2D, STE, and 3D assessment) and age at cancer diagnosis or duration of follow-up. Furthermore, no significant differences were found in echocardiographic findings when patients were divided according to sex, age at diagnosis <5 years, cumulative anthracycline dose >250 mg/m² and concomitant radiotherapy (data not shown).

## Discussion

A growing number of CCSs have to face lifelong side effects of cancer therapies, some of which affecting the cardiovascular system (3, 4, 6). Indeed, these patients have significant long-term morbidity related to their cancer therapy and cardiovascular events are the leading non-malignant cause of death. Compared with the general population, CCSs are at a 15-fold increased risk of developing congestive HF (19) and at a seven-fold increased risk of premature death due to cardiac causes (20). A variety of cardiovascular complications are increasingly appearing among CCSs, including dilated cardiomyopathy, myocardial infarction, valvular abnormalities, and pericarditis (21).

Among chemotherapeutics, anthracycline are widely used because of their high efficacy for treatment of childhood hematological malignancies and solid tumors, but are also those most commonly implicated in cardiac damage, resulting in an anthracycline-induced cardiomyopathy (22).

There is a strong dose-dependent relationship between anthracycline chemotherapy exposure and risk of congestive HF, with the incidence of cardiac dysfunction also increasing over time (4). Congestive HF and other adverse cardiovascular outcomes, indeed, may appear as late as 30 years after therapy (23). Of note, several studies have shown that cardiovascular complications may occur even after exposure to very low doses of the drug (23–25).

The cardiotoxicity of anthracycline may be acute, early or late (22). Acute cardiotoxicity occurs within the first week of anthracycline treatment; it is a transient depression of myocardial contractility and usually reversible when anthracycline are discontinued. Early onset cardiotoxicity occurs within the first year after the completion of anthracycline treatment; it results in dilated cardiomyopathy that can be a progressive disease. Late-onset cardiotoxicity occurs more than 1 year after the completion of anthracycline treatment and usually consists of dilated cardiomyopathy, which can show a progressive worsening over time.

Although characteristics and course of anthracycline-induced cardiotoxicity are still to be fully elucidated, the most commonly accepted pathophysiological mechanism is oxidative stress. According to this theory, the generation of reactive oxygen species and lipid peroxidation of the cell membrane can induce progressive cardiac remodeling as a late consequence of earlier myocyte injury, resulting in late cardiomyopathy (3).

Known risk factors for developing anthracycline-related cardiomyopathy include a cumulative dose higher than 250 mg/m^2^, infusion regimen, age younger than 5 years, additional radiation therapy, other concomitant chemotherapies and female sex (26). Other conditions seem to increase cardiac susceptibility, like hypertension and obesity; furthermore there are emerging data suggesting that genetic susceptibility could also have a role in modifying the individual response to therapeutic exposures (27, 28).

The time point when late cardiotoxicity becomes clinically manifest varies substantially. Many affected patients may initially be asymptomatic, with clinical manifestations appearing several years later, with a subsequent continuous progressive decline in myocardial function (3, 4). If anthracycline-associated cardiac dysfunction is detected early and treated with appropriate medications, patients frequently have a good functional recovery. Conversely, if patients are identified late after the onset of cardiac dysfunction, HF is typically more difficult to treat (5, 26, 29). For this reason, cardiac dysfunction should be detected as earlier as possible. This could be achieved through a tight long-term clinical and echocardiographic surveillance of these patients. However, the choice of surveillance modalities and recommendations for interventions are frequently based on expert consensus rather than trial data and depends upon local expertise and availability (30–32).

A recently published harmonization of international guidelines made an effort to provide surveillance recommendations for survivors of CCSs by an evidence-based approach and identifying subgroups at increased risk of developing cardiomyopathy (33).

Repeated echocardiographic assessment of LVEF remains the major way to identify a subclinical impairment of cardiac function. Conventional echocardiographic measurements of LV function, however, are not always adequate to detect early stages of cardiomyopathy (4, 9, 33). Importantly, this might be achieved using some new echocardiographic methods, that have been shown to be more sensitive at identifying early impairment of LV function (10).

The aim of this study was to assess which non-invasive echocardiography-based functional cardiac measures can detect early subclinical myocardial changes in a cohort of CCSs afferent to our institution.

### Two-Dimensional Echocardiography and Tissue Doppler Imaging

Traditionally, monitoring of anthracycline-related cardiotoxicity has relied on serial 2D echocardiography utilizing LVSF and LVEF (24, 34). However, these 2D Echo parameters are load-dependent, demonstrate intra-patient and inter-observer variability, and might not detect more subtle changes in cardiac systolic function (4, 9, 33). Our current study demonstrated significantly decreased LVSF in CCSs compared to healthy controls (p = 0.027), but no significant difference in LVEF, with all CCSs having LVSF and LVEF values within the normal range. Our findings are similar to those found in a recent multicenter cohort study by Slieker et al. (35), demonstrating lower measures of global systolic function in CCSs when compared with healthy controls; however, these differences were small and parameters of LV function were largely within the normal range. In contrast, another recent study by Border et al. (36) found a measurable longitudinal decline in many standard echocardiographic parameters of cardiac function, including FS and EF, in CCSs compared to controls, supporting the utility of conventional measures of global systolic function to detect myocardial dysfunction in cancer population.

These discordant results could be explained by differences in age and follow-up duration of CCSs across studies. For example, we investigated younger children compared to those of Border’s study, and this can be relevant, as it is well-known that advanced age and longer off-therapy time are important risk factors for the detection of more advanced disease. Therefore, two-dimensional LVEF likely may identify late stages of systolic dysfunction, whereas more sensitive methods are need for early detection of subclinical abnormalities of LV function.

An echo parameter designed to identify early myocardial disease is the MPI, which incorporates measures of systolic function (IVCT and LVET) and diastolic function (IVRT) and is independent of HR, ventricular geometry and preload/afterload (11). Previous reports supported the utility of MPI as an early marker of subclinical LV dysfunction in the surveillance of cardiotoxicity (34, 36–38). In our study, global myocardial function evaluated through MPI resulted significantly lower in cases than in controls (p = 0.005), with IVCT mildly increased (p = 0.04) and without significant differences in LVET and IVRT measures. At present time, even though MPI has been reported to identify early myocardial dysfunction, its clinical utility and prognostic value in progression of late anthracycline cardiomyopathy remains still unclear.

As a decrease in diastolic function often precedes the decline in systolic function (39), we investigated whether diastolic function assessment may provide a useful early marker of cardiac disease in CCSs patients. Diastolic dysfunction has been reported in several studies in CCSs exposed to anthracycline therapy (34, 36, 38, 40, 41) although Harahsheh et al. found normal diastolic function in these patients (42). In our study, we found mildly decreased E/A ratio in CCSs compared to controls (p = 0.046), but all diastolic parameters measured by pulsed wave Doppler and TDI were normal, without any significant difference between both groups. We observed an inverse correlation between the E/A ratio and the cumulative anthracycline dose. In clinical practice, surveillance of CCSs should include measures of diastolic function, although the utility of diastolic parameters to predict subsequent anthracycline-related cardiotoxicity warrants further follow-up studies.

### Speckle Tracking Imaging and 3D Echocardiography

Recently, myocardial strain imaging obtained by STE and 3D echo have been suggested to be more sensitive techniques than standard echocardiography for the earlier detection of subtle changes in myocardial function after anthracycline therapy (43). Recent reports from international Cardiological Societies recommend measurements of LV function by STE and 3D Echo in the long-term follow-up of adult cancer survivors (29, 44), but their role in pediatric CCSs has not been well established (33). Therefore, these advanced echocardiographic techniques require further validation with longitudinal follow-up studies, prior to be recommended in clinical practice, dependent also on availability and adequate operator experience (33, 35, 45).

Myocardial strain imaging, obtained by STE, evaluates the change in myocardial fiber length as compared to its original length in the plane in which it is measured (15). Unlike conventional LVSF and LVEF, which are limited to one plane, strain can be measured in three planes, providing a better determination of cardiac function. Previous studies provided evidence that strain abnormalities can be seen early despite preserved LVEF by conventional echo measures, identifying cardiac involvement in CCSs prior to the development of overt systolic dysfunction (43, 46–54). However, a more recent multicenter study of cardiac strain in a large cohort of pediatric CCSs found only mild differences in parameters of longitudinal strain between patients and healthy controls, with most CCSs (92.3%) having longitudinal strain values within the normal range. Thus, this report concluded that the utility of strain imaging in the long-term follow-up of CCSs remains to be demonstrated (35). Our study found normal values of global strain in both groups, and there was no significant difference in peak mean systolic longitudinal strain in any apical view. Furthermore, when stratifying for each segment, no difference was found in terms of regional longitudinal strain.

Among newer techniques, 3D echo has been shown to overcome the geometric limitations of 2D Echo for the assessment of LV volumes and EF and has been validated against CMR in assessing the LV volumes and EF in both children (17) and adults (18). In studies performed in survivors of childhood (9) and adult-onset cancer (55), 3D Echo demonstrated the lowest inter-observer and serial variability for measurement of LV systolic function, increasing the accuracy of detecting more subtle changes in LVEF. In this study, we found significant lower measures of three-dimensional LVEF in CCSs compared with healthy controls (p = 0.002), even though values were largely within the normal range. This finding of lower LVEF among CCSs using 3D Echo, agrees with several previous studies (9, 43, 47, 56, 57). Furthermore, several previously published studies found 3D Echo to have the highest sensitivity to detect more abnormalities in the ventricular function than conventional echocardiography, when compared to CMR (4, 9, 57).

In our study, we did not demonstrate statistically significant correlations between echocardiographic parameters (2D, strain and 3D assessment) and age at diagnosis or duration of follow-up. Similarly, a correlation between the echocardiographic parameters and established risk factors for anthracycline cardiomyopathy could not be documented. This may be due to a small sample size and/or young age of this cohort.

In conclusion, our study found significantly reduced 3D LVEF in CCSs compared with healthy controls, despite no significant differences in 2D EF and longitudinal strain values. These findings might support the use of 3D echo for the detection of early LVEF in CCSs.

## Conclusion

In recent years, cardiac surveillance for early detection of late subclinical cardiac dysfunction in CCSs treated with anthracycline has received increasing attention. Although commonly used, conventional echocardiographic parameters may not detect early cardiovascular damage in these patients. Novel imaging techniques, including ST imaging and 3D echo, have been investigated, but further longitudinal studies are necessary to clarify and optimize their role in routine clinical practice. The challenge with these non-conventional methods is to understand whether an abnormal result may be considered predictive of subsequent overt LV dysfunction, which might allow the identification of patients at risk and undertaking early preventive strategies.

### Limitations

Some limitations of our study should be acknowledged. First, we did not perform a preliminary sample size calculation to plan the number of subjects to enroll in the study. However, since previous studies showed that CCSs may have a decrease in LVEF of 10% (58, 59), we calculated that 6 subjects per group only might have been sufficient to have an 80% statistical power to detect as significant at two-tailed p < 0.05 a reduction of 10% in LVEF in CCS compared to healthy controls (LVEF 65.3 ± 4.4%). However, larger population may be required to better define the entity of LV impairment in CCSs. Second, CCSs were included among those referred to our University Center and therefore they may not reflect the general population of children treated with anthracyclines. Finally, the follow-up of our patients was done at 6 years from treatment and therefore we cannot exclude that some different results may be found at longer term.

## Data Availability Statement

The raw data supporting the conclusions of this article will be made available by the authors without undue reservation.

## Ethics Statement

Ethical review and approval was not required for the study on human participants in accordance with the local legislation and institutional requirements. Written informed consent from the participants’ legal guardian/next of kin was not required to participate in this study in accordance with the national legislation and the institutional requirements.

## Author Contributions

Conceptualization, AnR, AD, AL, and GL. Methodology, PL, VM, and AV. Writing—original draft preparation, AlR, RS, LB, and GA. Writing—review and editing, AD, RS and AL. Supervision, AnR and GL. Statistical analysis, GL, GA, and AV. All authors contributed to the article and approved the submitted version.

## Funding

This work was supported by Fondazione Policlinico Agostino Gemelli IRCCS.

## Conflict of Interest

The authors declare that the research was conducted in the absence of any commercial or financial relationships that could be construed as a potential conflict of interest.
